# Crystal structure of bis­(2-methyl-1*H*-imidazole-κ*N* ^3^)(*meso*-tetra-*p*-tol­ylporphyrinato-κ^4^ *N*)iron(III) perchlorate tetra­hydro­furan sesquisolvate

**DOI:** 10.1107/S2056989016010562

**Published:** 2016-07-12

**Authors:** Wenyan Sun, Jianfeng Li

**Affiliations:** a College of Materials Science and Optoelectronic Technology, University of Chinese Academy of Sciences, Yianqi Lake, Huairou District, Beijing, 101408, People’s Republic of China

**Keywords:** crystal structure, 2-methyl­imidazole, *meso*-tetra­kis­(*p*-tol­yl)porphyrinato, iron(III), highly ruffled porphyrin core, hydrogen bonding

## Abstract

In the title compound, [Fe(C_48_H_36_N_4_)(C_4_H_6_N_2_)_2_]ClO_4_·1.5C_4_H_8_O, the iron(III) metal is coordinated in a distorted octa­hedral geometry by four pyrrole N atoms of the porphyrin ligand in the equatorial plane and two N atoms of 2-methyl­imidazole ligands in the axial sites. The complex has a highly ruffled porphyrin core with mean absolute core-atom displacements *C*_a_, *C*_b_, *C*_m_ and *C*_av_ of 0.25 (5), 0.17 (12), 0.432 (16) and 0.25 (13) Å, respectively. One of the four phenyl groups of the porphyrin is disordered over two sets of sites with refined occupancy ratio of 0.718 (7):0.282 (7). The mean Fe—N_p_ (N_p_ is a porphyrin N atom) bond length [1.975 (9) Å] indicates the low-spin state of the iron atom. The two 2-methyl­imidazole ligands are nearly perpendicular and form a dihedral angle of 86.93 (10)°. The dihedral angles between the 2-methyl­imidazole ligands and the closest Fe—N_p_ vector are 38.04 (9) and 35.00 (7)°. In the crystal, the complex cations inter­act with the perchlorate anions through N—H⋯O hydrogen bonds, forming chains running parallel to [110].

## Abstract 2

The crystal structure of the six-coordinate, highly ruffled, ferric porphyrinate bis­(2-methyl­imidazole)[*meso*-tetra­kis­(*p*-tol­yl)porphyrinato]iron(III) perchlorate is reported.

## Chemical context   

The structural characterization of metalloporphyrin complexes with steric nitro­gen-donor ligands has been undertaken intensively in order to understand the control of structures, spin states, and other physical properties. Many structures of ferric porphyrins with general formula [Fe(Porph)(*L*)_2_]^+^ (Porph is a porphyrinato ligand and *L* is an N-bonded neutral ligand) and with the central Fe^III^ atom in an octahedral coordination are known. The first ferric porphyrin crystal structure with two sterically hindered axial ligands is [Fe(OEP)(2-MeHIm)_2_]ClO_4_, which was reported by Geiger and co-workers (Geiger *et al.*, 1984 ▸). Subsequently, some other analogues have been reported, [Fe(TPP)(2-MeHIm)_2_]ClO_4_ (Scheidt *et al.*, 1987 ▸), [Fe(TMP)(1,2-Me_2_Im)_2_]ClO_4_ (Munro *et al.*, 1995 ▸), [Fe(OETPP)(2-MeHIm)_2_]·(0.33SbF^6–^, 0.67Cl^−^) (Ogura *et al.*, 2001 ▸), [Fe(OMTPP)(2-MeHIm)_2_]Cl·3CD_2_Cl_2_ (Yatsunyk *et al.*, 2003 ▸), [Fe(OMTPP)(2-MeHIm)_2_]Cl·2CDCl_3_ (Yatsunyk *et al.*, 2003 ▸), *perp*-[Fe(OEP)(2-MeHIm)_2_]Cl (Hu *et al.*, 2006 ▸) (OEP, octa­ethyl­porphirin; TPP, tetra­phenyl­porphphyrin; TMP, tetra­mesitylporphyrin; OETPP, octa­ethyl­tetra­phenyl­porphyrin; OMTPP, octa­methyl­tetraphenylporphyrin; 2-MeHIm, 2-methyl­imidazole; 1,2-Me_2_Im, 1,2-di­methyl­imidazole). Herein, we report the structural properties of the iron(III) porphyrin complex [Fe(TTP)(2-MeHIm)_2_](ClO_4_)·1.5THF where the metal is likewise octahedrally coordinated.
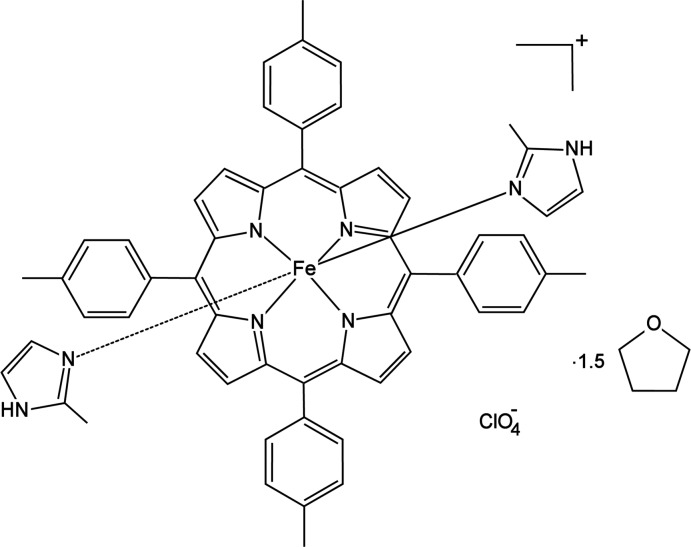


## Structural commentary   

**Figure 1:**
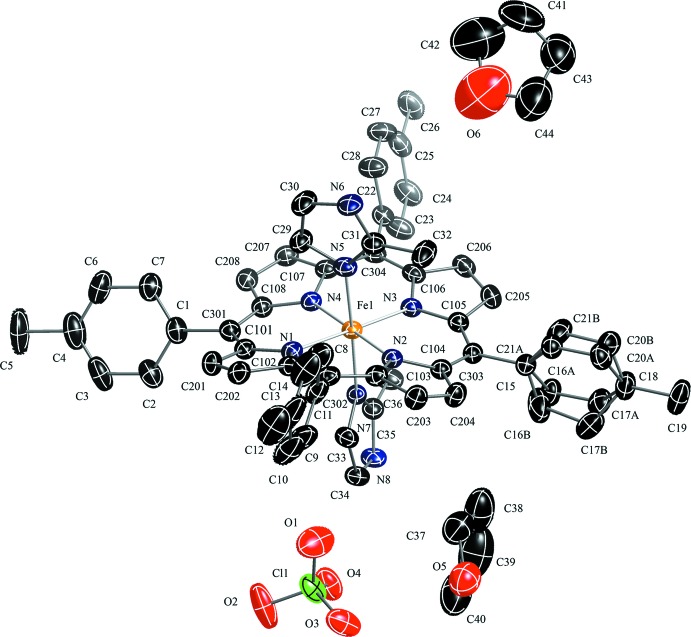
The mol­ecular structure of the title compound with displacement ellipsoids drawn at the 50% probability level. H atoms are omitted for clarity.

**Figure 2:**
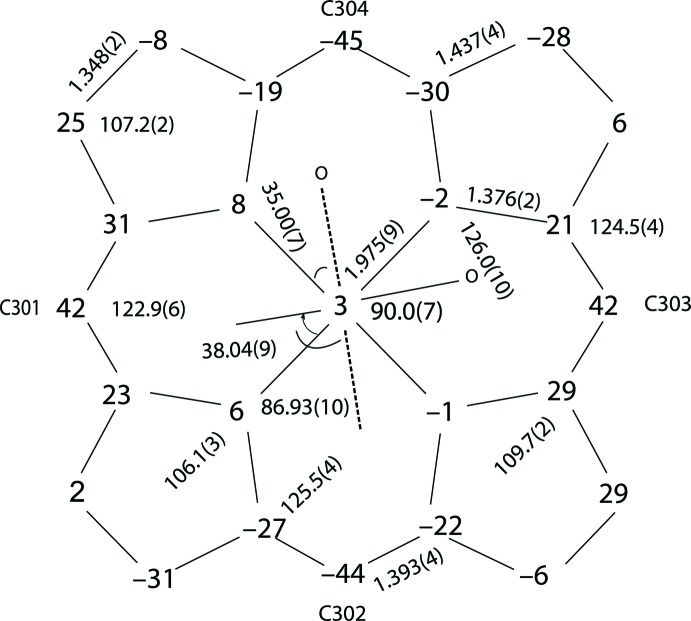
Diagram of the porphyrinato core of the title compound. Mean values of the chemically unique bond lengths (in Å) and angles (in °) are shown. The numbers in parentheses are the s.u. calculated on the assumption that the averaged values are all drawn from the same population. The perpendicular displacements (in units of 0.01 Å) of the porphyrin core atoms from the 24-atom mean plane are also displayed. Positive values indicate a displacement toward the N7 2-methyl­imidazole nitro­gen atom. The solid line in this perspective indicates the 2-methyl­imidazole ligand containing atom N7, and the dashed line indicates the 2-methyl­imidazole ligand containing atom N5. The small circle represents the position of the methyl group on the axial ligand.

In the title compound (Fig. 1 ▸), the counter-ion to the positively charged bis­(2-methyl­imidazole)[*meso*-tetra­kis­(*p*-tol­yl)porph­yrinato]iron(III) is a negatively charged perchlorate ion. One of the four phenyl groups of the porphyrin is disordered over two sets of sites [0.718 (7):0.282 (7)] and the dihedral angles between the disordered phenyl planes and the 24-atom mean plane are 72.4 (4) and 63.36 (12)°. Additional qu­anti­tative information on the structure is given in Fig. 2 ▸, which displays the detailed displacements of each porphyrin core atom from the 24-atom mean plane (in units of 0.01 Å). The mean values of the chemically unique bond lengths (in Å) and angles (in degrees), the orientations of the two 2-methyl­imidazole ligands including the values of the dihedral angles are also shown; the circle represents the position of the methyl group on the axial ligand. As indicated in Fig. 2 ▸, the 2-methyl­imidazole ligand containing the N7 atom makes a dihedral angle of 38.04 (9)°, the other making an angle of 35.00 (7)°, to the closest Fe—N_p_ vector. The relative orientation of the two 2-methyl­imidazole planes is nearly perpendicular, the dihedral angle being 86.93 (10)°. Fig. 2 ▸ also shows that the title complex has a highly ruffled porphyrin core conformation. The mean absolute core atom displacements of *C*_a_, *C*_b_, *C*_m_, and *C*_av_ are 0.25 (5), 0.17 (12), 0.432 (16) and 0.25 (13) Å, respectively. The mean Fe—N_p_ (N_p_ is a porphyrin N atom) bond length is 1.975 (9) Å, similar to 1.974 (4) Å in (*perp*-[Fe(OEP)(2-MeHIm)_2_]Cl) (Hu *et al.*, 2006 ▸) and 1.970 (4) Å in [Fe(TPP)(2-MeHIm)_2_]ClO_4_ (Scheidt *et al.*, 1987 ▸). These values are slightly shorter than 1.990 Å, which is typically observed for a low-spin iron(III) porphyrin complex (Scheidt & Reed, 1981 ▸).

The dihedral angles between the mean planes of the phenyl rings and the 24-atom mean plane are 59.55 (6), 82.53 (7), 72.4 (4) [and/or 63.36 (12)] and 75.17 (5)°, smaller than the same angles of 89.7, 83.3, 87.2 and 87.9° in [Fe(TMP)(1,2-Me_2_Im)_2_]ClO_4_ (Munro *et al.*, 1995 ▸). The reason for the difference could be the steric effect of the mesityl groups of [Fe(TMP)(1,2-Me_2_Im)_2_]ClO_4_, which hinders the rotation of the benzene groups.

## Supra­molecular features   

**Figure 3:**
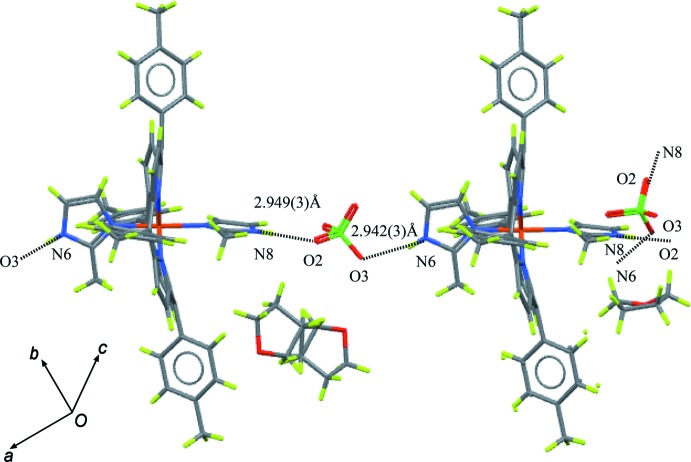
Partial packing diagram of the title compound showing the formation of a chain through hydrogen bonding between the perchlorate ion and two imidazole ligands. Dashed lines represent the hydrogen bonds. The O2⋯N8 and O3⋯N6 separations are given.

**Figure 4:**
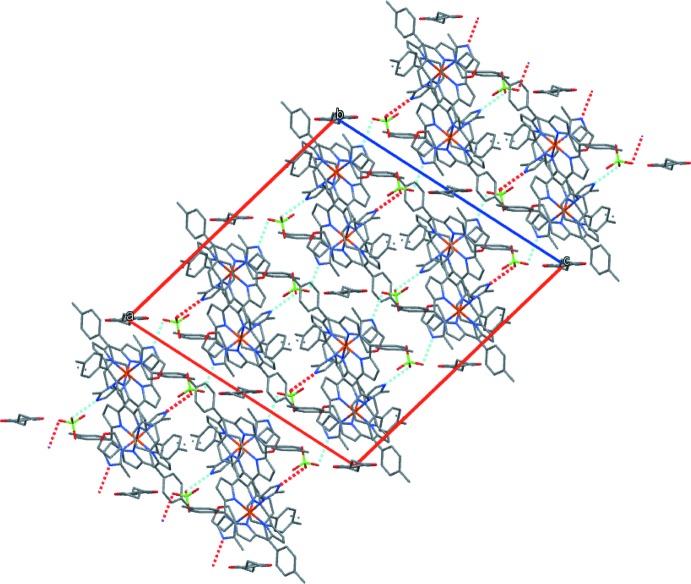
Packing diagram of the title compound viewed along the *a* axis, showing N—H⋯O hydrogen-bonding inter­actions as dashed lines. The disordered tetra­hydro­furan mol­ecules occupy the channels between the [Fe(TTP)(2-MeHIm)_2_]ClO_4_ mol­ecules. All H atoms are omitted.

N—H⋯O hydrogen bonds are observed in the crystal structure of the title compound (Table 1 ▸). As shown in Fig. 3 ▸, the perchlorate ion bridges two adjacent porphyrin mol­ecules through hydrogen bonding with imidazole ligands, which can be formulated as N8—H8*A*⋯O2—ClO_2_—O3⋯H6*A*—N6, forming a chain parallel to [110]. The hydrogen-bonding distances, 2.942 (3) (O3⋯N6) and 2.949 (3) Å (O2⋯N8), are consistent with the reported values 2.92 or 3.08 Å (Scheidt *et al.*, 1987 ▸; Hu *et al.*, 2006 ▸), and fall in the range 2.70–3.30 Å reported for inter­molecular N⋯O inter­actions (Bertolasi *et al.*, 1995 ▸). It is noteworthy that one of the tetra­hydro­furan mol­ecules, which is disordered about an inversion center, occupies the channels between the [Fe(TTP)(2-MeHIm)_2_]ClO_4_ complex mol­ecules (Fig. 4 ▸).

## Synthesis and crystallization   

**General Procedure**: All reactions were carried out using standard Schlenk techniques under argon unless otherwise noted. Tetra­hydro­furan (THF) and hexa­nes were distilled from sodium and benzo­phenone ketyl. H_2_(TTP) and [Fe(TTP)Cl] were prepared according to the reported methods (Adler *et al.*, 1970 ▸; Fleischer *et al.*, 1971 ▸).

### Synthesis of [*meso*-tetra­kis­(*p*-tol­yl)porphyrinato]iron(III) perchlorate   

[Fe(TTP)Cl] (500 mg, 0.652 mmol) and AgClO_4_ (136 mg, 0.657 mmol) were dissolved in 50 mL THF. After 12 h reaction, the solution was filtered and then evaporated to dryness under vacuum. The resulting purple solid, [Fe(TTP)ClO_4_], was harvested that was dried *in vacuo* (531.54 mg; yield 100%). UV–vis (CH_2_Cl_2_): 411.89, 516.5 nm.

### Synthesis of bis­(2-methyl-1*H*-imidazole-κ*N* ^3^)(*meso*-tetra-*p*-tol­ylporphyrinato-κ^4^ *N*)iron(III) perchlorate tetra­hydro­furan sesquisolvate   

[Fe(TTP)ClO_4_] (20 mg, 0.024 mmol) and excess 2-methyl­imidazole (0.164 g, 2 mmol) were dissolved in 7 mL THF. After 10 min, the solution was transferred into glass tubes which were layered with hexa­nes as nonsolvent. Dark-purple block-shaped crystals suitable for a single-crystal X-ray diffraction study were collected after 15 d. UV–vis (CH_2_Cl_2_): 415.44, 509.68, 571.87, 612.00 nm.

## Refinement   

Crystal data, data collection and structure refinement details are summarized in Table 2 ▸. The hydrogen atoms of the two imidazole nitro­gen atoms of the axial ligands were located in a difference Fourier map and refined freely. All other hydrogen atoms were placed in calculated positions, with C—H = 0.95 or 0.98 Å for aryl or methyl H atoms, respectively, and refined using a riding model with *U*_iso_(H) = 1.5*U*_eq_(C) for methyl H atoms or *U*_iso_(H) = 1.2*U*_eq_(C, N) otherwise. One THF mol­ecule is disordered over two sets of sites about an inversion center with an occupancy factor of 0.5. During the refinement, the O—C, C—C and C⋯C distances within the disordered THF mol­ecule were constrained to be 1.42 (1), 1.50 (1) and 2.40 (1) Å, respectively. One of the four phenyl groups was found to be disordered over two orientations and the site occupancy factors (SOFs) of disordered moieties are refined by means of a ‘free variable’. The refined final SOFs were 0.718 (7) and 0.282 (7). Two carbon atoms (C39 and C43) of the tetra­hydro­furan mol­ecules and one carbon atom (C12) of a methyl group exhibited unusually large displacement parameters and thus were refined using SIMU and ISOR restraints. Seven outliers were omitted in the last cycles of refinement.

## Supplementary Material

Crystal structure: contains datablock(s) I. DOI: 10.1107/S2056989016010562/rz5191sup1.cif

Structure factors: contains datablock(s) I. DOI: 10.1107/S2056989016010562/rz5191Isup2.hkl

CCDC reference: 1471779

Additional supporting information: crystallographic information; 3D view; checkCIF report

## Figures and Tables

**Table 1:** Hydrogen-bond geometry (Å, °)

*D*—H⋯*A*	*D*—H	H⋯*A*	*D*⋯*A*	*D*—H⋯*A*
N6—H6*A*⋯O3^i^	0.81 (3)	2.17 (3)	2.942 (3)	161 (3)
N8—H8*A*⋯O2^ii^	0.84 (3)	2.11 (3)	2.949 (3)	176 (3)

Symmetry codes: (i) 
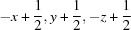
; (ii) 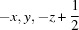.

**Table 2:** Experimental details

Crystal data	
Chemical formula	[Fe(C_48_H_36_N_4_)(C_4_H_6_N_2_)_2_]ClO_4_·1.5C_4_H_8_O
*M*_r_	1096.48
Crystal system, space group	Monoclinic, *C*2/*c*
Temperature (K)	130
*a*, *b*, *c* (Å)	26.7161 (10), 16.6111 (6), 24.9673 (8)
β (°)	103.538 (1)
*V* (Å^3^)	10772.2 (7)
*Z*	8
Radiation type	Mo *K*α
μ (mm^−1^)	0.39
Crystal size (mm)	0.52 × 0.23 × 0.20

Data collection	
Diffractometer	Brucker D8 QUEST System
Absorption correction	Multi-scan (*SADABS*; Bruker, 2014 ▸)
*T*_min_, *T*_max_	0.898, 0.925
No. of measured, independent and observed [*I* > 2σ(*I*)] reflections	120242, 11928, 9751
*R*_int_	0.051
(sin θ/λ)_max_ (Å^−1^)	0.642

Refinement	
*R*[*F* ^2^ > 2σ(*F* ^2^)], *wR*(*F* ^2^), *S*	0.048, 0.133, 1.04
No. of reflections	11928
No. of parameters	772
No. of restraints	64
H-atom treatment	H atoms treated by a mixture of independent and constrained refinement
Δρ_max_, Δρ_min_ (e Å^−3^)	0.77, −0.79

Computer programs: *APEX2* and *SAINT-Plus* (Bruker, 2014 ▸), *SHELXT2014* (Sheldrick, 2015*a* ▸), *SHELXL2014/6* (Sheldrick, 2015*b* ▸), *SHELXTL* (Sheldrick, 2008 ▸), *Mercury* (Macrae *et al.*, 2006 ▸) and *publCIF* (Westrip, 2010 ▸).

## supplementary crystallographic information

### Crystal data

**Table:**

[Fe(C_48_H_36_N_4_)(C_4_H_6_N_2_)_2_]ClO_4_·1.5C_4_H_8_O	*F*(000) = 4600
*M**_r_* = 1096.48	*D*_x_ = 1.352 Mg m^−^^3^
Monoclinic, *C*2/*c*	Mo *K*α radiation, λ = 0.71073 Å
*a* = 26.7161 (10) Å	Cell parameters from 9943 reflections
*b* = 16.6111 (6) Å	θ = 2.5–27.1°
*c* = 24.9673 (8) Å	µ = 0.39 mm^−^^1^
β = 103.538 (1)°	*T* = 130 K
*V* = 10772.2 (7) Å^3^	Block, dark purple
*Z* = 8	0.52 × 0.23 × 0.20 mm

### Data collection

**Table:**

Brucker D8 QUEST System diffractometer	11928 independent reflections
Radiation source: fine-focus sealed tube	9751 reflections with *I* > 2σ(*I*)
Detector resolution: 0 pixels mm^-1^	*R*_int_ = 0.051
φ and ω scans	θ_max_ = 27.2°, θ_min_ = 2.4°
Absorption correction: multi-scan (SADABS; Bruker, 2014)	*h* = −34→34
*T*_min_ = 0.898, *T*_max_ = 0.925	*k* = −21→21
120242 measured reflections	*l* = −32→31

### Refinement

**Table:**

Refinement on *F*^2^	Primary atom site location: structure-invariant direct methods
Least-squares matrix: full	Secondary atom site location: difference Fourier map
*R*[*F*^2^ > 2σ(*F*^2^)] = 0.048	Hydrogen site location: mixed
*wR*(*F*^2^) = 0.133	H atoms treated by a mixture of independent and constrained refinement
*S* = 1.04	*w* = 1/[σ^2^(*F*_o_^2^) + (0.0586*P*)^2^ + 27.1309*P*] where *P* = (*F*_o_^2^ + 2*F*_c_^2^)/3
11928 reflections	(Δ/σ)_max_ < 0.001
772 parameters	Δρ_max_ = 0.77 e Å^−^^3^
64 restraints	Δρ_min_ = −0.79 e Å^−^^3^

### Special details

**Table:**

Geometry. All esds (except the esd in the dihedral angle between two l.s. planes) are estimated using the full covariance matrix. The cell esds are taken into account individually in the estimation of esds in distances, angles and torsion angles; correlations between esds in cell parameters are only used when they are defined by crystal symmetry. An approximate (isotropic) treatment of cell esds is used for estimating esds involving l.s. planes.
Refinement. Refinement of F^2^ against ALL reflections. The weighted R-factor wR and goodness of fit S are based on F^2^, conventional R-factors R are based on F, with F set to zero for negative F^2^. The threshold expression of F^2^ > 2sigma(F^2^) is used only for calculating R-factors(gt) etc. and is not relevant to the choice of reflections for refinement. R-factors based on F^2^ are statistically about twice as large as those based on F, and R- factors based on ALL data will be even larger.

### Fractional atomic coordinates and isotropic or equivalent isotropic displacement parameters (Å^2^)

**Table:**

	*x*	*y*	*z*	*U*_iso_*/*U*_eq_	Occ. (<1)
Fe1	0.16104 (2)	0.38552 (2)	0.14780 (2)	0.01685 (8)	
N1	0.17087 (7)	0.42281 (10)	0.22511 (7)	0.0204 (3)	
N2	0.20284 (6)	0.28919 (10)	0.17281 (7)	0.0189 (3)	
N3	0.15186 (6)	0.34956 (10)	0.07103 (7)	0.0189 (3)	
N4	0.11862 (6)	0.48227 (10)	0.12318 (7)	0.0193 (3)	
N5	0.22345 (7)	0.45107 (11)	0.14473 (7)	0.0233 (4)	
N6	0.29935 (8)	0.49768 (13)	0.14308 (9)	0.0322 (5)	
N7	0.10050 (6)	0.31950 (10)	0.15599 (7)	0.0195 (3)	
N8	0.03203 (7)	0.24413 (12)	0.14613 (8)	0.0269 (4)	
C1	0.09287 (9)	0.60715 (15)	0.24183 (9)	0.0288 (5)	
C2	0.06470 (10)	0.59122 (18)	0.28095 (10)	0.0385 (6)	
H2A	0.0548	0.5375	0.2864	0.046*	
C3	0.05095 (11)	0.6531 (2)	0.31211 (11)	0.0464 (7)	
H3A	0.0319	0.6409	0.3388	0.056*	
C4	0.06434 (12)	0.7319 (2)	0.30512 (12)	0.0489 (7)	
C5	0.04932 (16)	0.7986 (2)	0.33998 (15)	0.0738 (12)	
H5A	0.0588	0.7830	0.3789	0.111*	
H5B	0.0121	0.8075	0.3288	0.111*	
H5C	0.0674	0.8483	0.3347	0.111*	
C6	0.09204 (12)	0.74830 (18)	0.26575 (13)	0.0477 (7)	
H6B	0.1016	0.8022	0.2602	0.057*	
C7	0.10602 (10)	0.68681 (15)	0.23438 (11)	0.0357 (5)	
H7A	0.1248	0.6993	0.2075	0.043*	
C8	0.27637 (10)	0.30770 (14)	0.32177 (9)	0.0308 (5)	
C9	0.26168 (14)	0.27067 (17)	0.36579 (11)	0.0493 (8)	
H9A	0.2270	0.2543	0.3624	0.059*	
C10	0.29832 (18)	0.25788 (18)	0.41492 (12)	0.0673 (11)	
H10A	0.2884	0.2316	0.4446	0.081*	
C11	0.34866 (17)	0.28270 (18)	0.42120 (13)	0.0652 (11)	
C12	0.38845 (17)	0.2746 (3)	0.47557 (16)	0.0794 (11)	
H12A	0.3803	0.2276	0.4956	0.119*	
H12B	0.3880	0.3232	0.4978	0.119*	
H12C	0.4227	0.2679	0.4683	0.119*	
C13	0.36259 (14)	0.3182 (2)	0.37762 (14)	0.0584 (9)	
H13A	0.3973	0.3345	0.3811	0.070*	
C14	0.32659 (11)	0.33096 (18)	0.32803 (11)	0.0433 (6)	
H14A	0.3370	0.3561	0.2982	0.052*	
C15	0.19393 (9)	0.13309 (13)	0.06029 (9)	0.0238 (4)	
C16A	0.15291 (14)	0.0846 (2)	0.03279 (15)	0.0298 (9)	0.718 (7)
H16A	0.1184	0.1006	0.0309	0.036*	0.718 (7)
C17A	0.16285 (14)	0.0133 (2)	0.00834 (16)	0.0342 (10)	0.718 (7)
H17A	0.1347	−0.0188	−0.0104	0.041*	0.718 (7)
C20A	0.25309 (17)	0.0380 (2)	0.0357 (2)	0.0334 (9)	0.718 (7)
H20A	0.2874	0.0230	0.0357	0.040*	0.718 (7)
C21A	0.24418 (15)	0.1092 (2)	0.06081 (19)	0.0286 (8)	0.718 (7)
H21A	0.2724	0.1420	0.0785	0.034*	0.718 (7)
C16B	0.1670 (6)	0.0762 (6)	0.0652 (5)	0.068 (4)	0.282 (7)
H16B	0.1376	0.0853	0.0797	0.082*	0.282 (7)
C17B	0.1782 (5)	−0.0104 (7)	0.0492 (6)	0.055 (4)	0.282 (7)
H17B	0.1648	−0.0571	0.0629	0.066*	0.282 (7)
C20B	0.2370 (5)	0.0501 (7)	0.0056 (7)	0.044 (3)	0.282 (7)
H20B	0.2628	0.0473	−0.0149	0.052*	0.282 (7)
C21B	0.2286 (5)	0.1243 (7)	0.0294 (7)	0.045 (3)	0.282 (7)
H21B	0.2482	0.1698	0.0234	0.054*	0.282 (7)
C18	0.21214 (11)	−0.01254 (15)	0.01033 (10)	0.0361 (6)	
C19	0.22246 (13)	−0.09109 (18)	−0.01582 (14)	0.0525 (8)	
H19A	0.1899	−0.1134	−0.0372	0.079*	
H19B	0.2384	−0.1293	0.0130	0.079*	
H19C	0.2457	−0.0814	−0.0402	0.079*	
C22	0.09844 (8)	0.51590 (13)	−0.03016 (9)	0.0234 (4)	
C23	0.05332 (9)	0.49667 (16)	−0.06809 (10)	0.0328 (5)	
H23A	0.0317	0.4553	−0.0599	0.039*	
C24	0.03932 (10)	0.53730 (18)	−0.11795 (10)	0.0380 (6)	
H24A	0.0081	0.5236	−0.1433	0.046*	
C25	0.06998 (10)	0.59735 (15)	−0.13145 (10)	0.0335 (5)	
C26	0.05609 (14)	0.63851 (18)	−0.18665 (11)	0.0485 (7)	
H26A	0.0187	0.6364	−0.2012	0.073*	
H26B	0.0732	0.6112	−0.2122	0.073*	
H26C	0.0673	0.6948	−0.1825	0.073*	
C27	0.11408 (11)	0.61758 (16)	−0.09343 (11)	0.0401 (6)	
H27A	0.1354	0.6594	−0.1016	0.048*	
C28	0.12813 (10)	0.57798 (16)	−0.04309 (10)	0.0360 (6)	
H28A	0.1586	0.5937	−0.0172	0.043*	
C29	0.22672 (9)	0.53233 (14)	0.16050 (11)	0.0329 (5)	
H29A	0.2000	0.5626	0.1702	0.039*	
C30	0.27349 (10)	0.56053 (16)	0.15976 (12)	0.0400 (6)	
H30A	0.2862	0.6134	0.1689	0.048*	
C31	0.26867 (9)	0.43286 (15)	0.13458 (10)	0.0296 (5)	
C32	0.28581 (11)	0.35627 (17)	0.11542 (15)	0.0506 (8)	
H32A	0.3169	0.3654	0.1018	0.076*	
H32B	0.2585	0.3345	0.0857	0.076*	
H32C	0.2935	0.3179	0.1461	0.076*	
C33	0.10220 (8)	0.27606 (13)	0.20441 (9)	0.0250 (4)	
H33A	0.1292	0.2787	0.2368	0.030*	
C34	0.05993 (9)	0.23006 (14)	0.19819 (9)	0.0285 (5)	
H34A	0.0513	0.1950	0.2247	0.034*	
C35	0.05671 (8)	0.29830 (13)	0.12129 (9)	0.0241 (4)	
C36	0.03539 (9)	0.32524 (16)	0.06396 (10)	0.0326 (5)	
H36A	−0.0015	0.3129	0.0533	0.049*	
H36B	0.0531	0.2973	0.0392	0.049*	
H36C	0.0404	0.3834	0.0614	0.049*	
C101	0.14767 (8)	0.48710 (13)	0.24429 (9)	0.0225 (4)	
C102	0.20771 (8)	0.39485 (13)	0.26924 (8)	0.0222 (4)	
C103	0.23263 (8)	0.27496 (13)	0.22463 (9)	0.0229 (4)	
C104	0.20483 (8)	0.21955 (13)	0.14336 (9)	0.0213 (4)	
C105	0.16228 (8)	0.27430 (13)	0.05314 (8)	0.0210 (4)	
C106	0.13440 (8)	0.39558 (13)	0.02454 (8)	0.0209 (4)	
C107	0.10441 (8)	0.51038 (13)	0.06986 (8)	0.0210 (4)	
C108	0.09999 (8)	0.53740 (13)	0.15480 (9)	0.0219 (4)	
C201	0.17010 (9)	0.49869 (14)	0.30216 (9)	0.0276 (5)	
H20I	0.1603	0.5380	0.3254	0.033*	
C202	0.20757 (9)	0.44333 (14)	0.31710 (9)	0.0276 (5)	
H20J	0.2298	0.4373	0.3526	0.033*	
C203	0.25371 (9)	0.19535 (14)	0.22771 (9)	0.0289 (5)	
H20C	0.2763	0.1716	0.2588	0.035*	
C204	0.23555 (9)	0.16046 (14)	0.17827 (9)	0.0286 (5)	
H20D	0.2419	0.1068	0.1684	0.034*	
C205	0.15073 (9)	0.27346 (14)	−0.00599 (9)	0.0267 (5)	
H20E	0.1537	0.2285	−0.0286	0.032*	
C206	0.13488 (9)	0.34813 (14)	−0.02363 (9)	0.0260 (5)	
H20F	0.1258	0.3660	−0.0608	0.031*	
C207	0.07487 (8)	0.58290 (13)	0.06819 (9)	0.0250 (4)	
H20G	0.0605	0.6140	0.0363	0.030*	
C208	0.07116 (8)	0.59883 (13)	0.12012 (9)	0.0252 (4)	
H20H	0.0530	0.6423	0.1316	0.030*	
C301	0.11206 (8)	0.53968 (13)	0.21232 (9)	0.0234 (4)	
C302	0.23792 (8)	0.32704 (13)	0.26953 (9)	0.0236 (4)	
C303	0.18481 (8)	0.21076 (13)	0.08682 (9)	0.0216 (4)	
C304	0.11429 (8)	0.47294 (13)	0.02367 (8)	0.0215 (4)	
O6	0.4567 (5)	0.4345 (7)	0.0516 (5)	0.231 (4)	0.5
C41	0.5128 (6)	0.4673 (7)	−0.0064 (6)	0.134 (3)	0.5
H41A	0.5479	0.4634	0.0175	0.160*	0.5
H41B	0.5138	0.4987	−0.0398	0.160*	0.5
C42	0.4765 (9)	0.5015 (9)	0.0230 (9)	0.199 (5)	0.5
H42A	0.4478	0.5282	−0.0032	0.239*	0.5
H42B	0.4940	0.5419	0.0501	0.239*	0.5
C43	0.4895 (5)	0.3869 (6)	−0.0203 (5)	0.117 (3)	0.5
H43A	0.4585	0.3903	−0.0510	0.140*	0.5
H43B	0.5144	0.3492	−0.0305	0.140*	0.5
C44	0.4763 (6)	0.3621 (7)	0.0306 (5)	0.140 (4)	0.5
H44A	0.5070	0.3416	0.0573	0.168*	0.5
H44B	0.4497	0.3194	0.0233	0.168*	0.5
Cl1	0.08673 (2)	0.12887 (4)	0.35815 (3)	0.03735 (15)	
O1	0.13065 (9)	0.16939 (16)	0.34826 (14)	0.0786 (8)	
O2	0.06869 (9)	0.16899 (17)	0.40094 (8)	0.0667 (7)	
O3	0.09973 (8)	0.04691 (14)	0.37399 (9)	0.0543 (6)	
O4	0.04620 (8)	0.13067 (13)	0.30954 (8)	0.0477 (5)	
O5	0.11480 (11)	−0.04122 (16)	0.24469 (12)	0.0730 (7)	
C37	0.13725 (18)	0.0233 (2)	0.22114 (18)	0.0760 (11)	
H37A	0.1736	0.0110	0.2215	0.091*	
H37B	0.1361	0.0735	0.2422	0.091*	
C38	0.1065 (2)	0.0324 (3)	0.16355 (19)	0.0967 (16)	
H38A	0.1219	0.0010	0.1378	0.116*	
H38B	0.1044	0.0896	0.1522	0.116*	
C39	0.0546 (2)	0.0006 (4)	0.1646 (3)	0.119 (2)	
H39A	0.0444	−0.0433	0.1375	0.143*	
H39B	0.0284	0.0438	0.1565	0.143*	
C40	0.06130 (17)	−0.0306 (3)	0.2235 (2)	0.0971 (16)	
H40A	0.0474	0.0086	0.2461	0.117*	
H40B	0.0429	−0.0824	0.2235	0.117*	
H6A	0.3286 (13)	0.4996 (19)	0.1394 (13)	0.046 (9)*	
H8A	0.0030 (12)	0.2249 (18)	0.1317 (12)	0.037 (8)*	

### Atomic displacement parameters (Å^2^)

**Table:**

	*U*^11^	*U*^22^	*U*^33^	*U*^12^	*U*^13^	*U*^23^
Fe1	0.01546 (14)	0.01825 (15)	0.01730 (14)	−0.00454 (11)	0.00480 (10)	−0.00147 (11)
N1	0.0208 (8)	0.0196 (8)	0.0209 (8)	−0.0045 (7)	0.0048 (7)	−0.0015 (7)
N2	0.0190 (8)	0.0195 (8)	0.0191 (8)	−0.0034 (7)	0.0060 (6)	−0.0007 (6)
N3	0.0175 (8)	0.0203 (8)	0.0196 (8)	−0.0032 (7)	0.0061 (6)	−0.0006 (7)
N4	0.0184 (8)	0.0198 (8)	0.0203 (8)	−0.0045 (7)	0.0054 (6)	−0.0019 (7)
N5	0.0202 (9)	0.0260 (9)	0.0236 (9)	−0.0063 (7)	0.0051 (7)	0.0001 (7)
N6	0.0204 (9)	0.0323 (11)	0.0463 (12)	−0.0104 (8)	0.0130 (9)	−0.0049 (9)
N7	0.0190 (8)	0.0189 (8)	0.0221 (8)	−0.0032 (7)	0.0076 (7)	−0.0031 (7)
N8	0.0200 (9)	0.0321 (10)	0.0294 (10)	−0.0113 (8)	0.0077 (8)	−0.0020 (8)
C1	0.0247 (11)	0.0336 (12)	0.0270 (11)	0.0020 (9)	0.0038 (9)	−0.0057 (9)
C2	0.0366 (14)	0.0484 (16)	0.0324 (13)	−0.0008 (12)	0.0118 (11)	−0.0074 (11)
C3	0.0407 (15)	0.067 (2)	0.0346 (14)	0.0104 (14)	0.0146 (12)	−0.0109 (13)
C4	0.0448 (16)	0.0569 (19)	0.0408 (15)	0.0180 (14)	0.0015 (12)	−0.0170 (14)
C5	0.080 (3)	0.077 (3)	0.061 (2)	0.037 (2)	0.0098 (19)	−0.0297 (19)
C6	0.0502 (17)	0.0385 (15)	0.0510 (17)	0.0099 (13)	0.0050 (13)	−0.0124 (13)
C7	0.0342 (13)	0.0328 (13)	0.0397 (14)	0.0032 (10)	0.0078 (11)	−0.0066 (11)
C8	0.0437 (14)	0.0204 (11)	0.0234 (11)	0.0001 (10)	−0.0023 (10)	−0.0018 (9)
C9	0.078 (2)	0.0335 (14)	0.0315 (14)	−0.0141 (14)	0.0033 (14)	0.0050 (11)
C10	0.131 (4)	0.0319 (15)	0.0289 (14)	−0.0111 (19)	−0.0017 (18)	0.0085 (12)
C11	0.108 (3)	0.0256 (14)	0.0394 (16)	0.0038 (17)	−0.0291 (18)	−0.0003 (12)
C12	0.0912 (19)	0.0646 (17)	0.0625 (16)	0.0036 (15)	−0.0224 (14)	−0.0008 (14)
C13	0.0556 (19)	0.0475 (18)	0.0554 (19)	0.0009 (15)	−0.0206 (15)	−0.0040 (15)
C14	0.0411 (15)	0.0450 (16)	0.0360 (14)	−0.0022 (12)	−0.0065 (11)	0.0018 (12)
C15	0.0283 (11)	0.0207 (10)	0.0236 (10)	−0.0013 (9)	0.0085 (9)	−0.0019 (8)
C16A	0.0308 (18)	0.0303 (18)	0.0253 (18)	−0.0022 (14)	0.0007 (14)	−0.0087 (14)
C17A	0.0368 (19)	0.0274 (18)	0.036 (2)	−0.0046 (14)	0.0042 (15)	−0.0106 (15)
C20A	0.034 (2)	0.032 (2)	0.037 (2)	0.0063 (16)	0.0129 (18)	−0.0034 (18)
C21A	0.0280 (18)	0.0273 (18)	0.032 (2)	0.0005 (14)	0.0092 (16)	−0.0044 (15)
C16B	0.054 (6)	0.081 (7)	0.080 (8)	−0.006 (6)	0.037 (6)	−0.049 (6)
C17B	0.069 (8)	0.034 (6)	0.067 (9)	−0.011 (5)	0.023 (7)	−0.007 (5)
C20B	0.042 (6)	0.036 (6)	0.062 (9)	0.008 (5)	0.029 (6)	−0.004 (6)
C21B	0.037 (6)	0.030 (5)	0.075 (10)	−0.010 (4)	0.029 (6)	−0.012 (6)
C18	0.0474 (15)	0.0286 (12)	0.0319 (12)	0.0049 (11)	0.0085 (11)	−0.0069 (10)
C19	0.064 (2)	0.0363 (15)	0.0539 (18)	0.0110 (14)	0.0075 (15)	−0.0170 (13)
C22	0.0263 (11)	0.0247 (11)	0.0211 (10)	−0.0004 (8)	0.0092 (8)	0.0006 (8)
C23	0.0279 (12)	0.0428 (14)	0.0270 (11)	−0.0052 (10)	0.0052 (9)	0.0075 (10)
C24	0.0321 (13)	0.0541 (16)	0.0262 (12)	0.0029 (12)	0.0035 (10)	0.0065 (11)
C25	0.0461 (14)	0.0333 (13)	0.0247 (11)	0.0128 (11)	0.0161 (10)	0.0063 (10)
C26	0.076 (2)	0.0449 (16)	0.0288 (13)	0.0184 (15)	0.0200 (13)	0.0121 (12)
C27	0.0515 (16)	0.0359 (14)	0.0365 (14)	−0.0089 (12)	0.0180 (12)	0.0084 (11)
C28	0.0371 (13)	0.0372 (14)	0.0325 (12)	−0.0113 (11)	0.0057 (10)	0.0048 (10)
C29	0.0291 (12)	0.0267 (12)	0.0463 (14)	−0.0061 (9)	0.0161 (11)	−0.0071 (10)
C30	0.0337 (13)	0.0298 (13)	0.0603 (17)	−0.0117 (11)	0.0183 (12)	−0.0106 (12)
C31	0.0233 (11)	0.0312 (12)	0.0358 (12)	−0.0058 (9)	0.0102 (9)	−0.0029 (10)
C32	0.0365 (15)	0.0377 (15)	0.088 (2)	−0.0111 (12)	0.0349 (15)	−0.0188 (15)
C33	0.0256 (11)	0.0275 (11)	0.0242 (10)	−0.0042 (9)	0.0101 (8)	−0.0007 (9)
C34	0.0298 (11)	0.0301 (12)	0.0286 (11)	−0.0060 (9)	0.0131 (9)	0.0008 (9)
C35	0.0188 (10)	0.0263 (11)	0.0287 (11)	−0.0037 (8)	0.0083 (8)	−0.0041 (9)
C36	0.0232 (11)	0.0428 (14)	0.0305 (12)	−0.0082 (10)	0.0040 (9)	0.0026 (10)
C101	0.0227 (10)	0.0227 (10)	0.0228 (10)	−0.0056 (8)	0.0071 (8)	−0.0036 (8)
C102	0.0256 (10)	0.0227 (10)	0.0179 (9)	−0.0063 (8)	0.0041 (8)	−0.0016 (8)
C103	0.0223 (10)	0.0237 (10)	0.0218 (10)	−0.0024 (8)	0.0035 (8)	0.0001 (8)
C104	0.0200 (9)	0.0212 (10)	0.0235 (10)	−0.0021 (8)	0.0068 (8)	−0.0006 (8)
C105	0.0197 (9)	0.0230 (10)	0.0218 (10)	−0.0025 (8)	0.0079 (8)	−0.0023 (8)
C106	0.0190 (10)	0.0266 (11)	0.0182 (9)	−0.0060 (8)	0.0062 (8)	0.0001 (8)
C107	0.0185 (9)	0.0224 (10)	0.0220 (10)	−0.0049 (8)	0.0042 (8)	0.0009 (8)
C108	0.0191 (9)	0.0233 (10)	0.0238 (10)	−0.0032 (8)	0.0060 (8)	−0.0030 (8)
C201	0.0332 (12)	0.0266 (11)	0.0230 (11)	−0.0046 (9)	0.0067 (9)	−0.0072 (9)
C202	0.0342 (12)	0.0259 (11)	0.0210 (10)	−0.0068 (9)	0.0031 (9)	−0.0036 (9)
C203	0.0324 (12)	0.0261 (11)	0.0254 (11)	0.0019 (9)	0.0009 (9)	0.0012 (9)
C204	0.0331 (12)	0.0216 (11)	0.0301 (11)	0.0032 (9)	0.0056 (9)	−0.0011 (9)
C205	0.0315 (11)	0.0292 (11)	0.0208 (10)	−0.0012 (9)	0.0090 (9)	−0.0041 (9)
C206	0.0298 (11)	0.0305 (12)	0.0195 (10)	−0.0016 (9)	0.0094 (9)	−0.0004 (9)
C207	0.0232 (10)	0.0245 (11)	0.0266 (11)	−0.0020 (8)	0.0044 (8)	0.0024 (9)
C208	0.0218 (10)	0.0240 (11)	0.0296 (11)	0.0000 (8)	0.0059 (9)	−0.0019 (9)
C301	0.0202 (10)	0.0259 (11)	0.0253 (10)	−0.0030 (8)	0.0077 (8)	−0.0039 (8)
C302	0.0257 (11)	0.0227 (10)	0.0210 (10)	−0.0064 (8)	0.0024 (8)	0.0007 (8)
C303	0.0206 (10)	0.0211 (10)	0.0251 (10)	−0.0018 (8)	0.0093 (8)	−0.0032 (8)
C304	0.0191 (9)	0.0253 (10)	0.0202 (10)	−0.0052 (8)	0.0047 (8)	0.0015 (8)
O6	0.254 (9)	0.178 (8)	0.254 (9)	−0.026 (8)	0.043 (8)	−0.002 (7)
C41	0.152 (7)	0.122 (7)	0.163 (8)	−0.039 (6)	0.111 (6)	−0.001 (6)
C42	0.229 (9)	0.160 (9)	0.217 (10)	−0.040 (8)	0.073 (8)	0.014 (8)
C43	0.118 (6)	0.101 (5)	0.148 (6)	−0.010 (5)	0.065 (5)	−0.035 (5)
C44	0.144 (7)	0.107 (7)	0.185 (8)	−0.001 (6)	0.067 (6)	−0.051 (6)
Cl1	0.0258 (3)	0.0514 (4)	0.0370 (3)	0.0186 (3)	0.0116 (2)	0.0069 (3)
O1	0.0361 (12)	0.0641 (16)	0.144 (3)	0.0085 (11)	0.0388 (15)	0.0125 (16)
O2	0.0532 (13)	0.109 (2)	0.0331 (10)	0.0416 (14)	0.0008 (9)	−0.0140 (12)
O3	0.0407 (11)	0.0632 (14)	0.0648 (14)	0.0280 (10)	0.0242 (10)	0.0295 (11)
O4	0.0520 (12)	0.0594 (13)	0.0298 (9)	0.0218 (10)	0.0059 (8)	0.0043 (9)
O5	0.0675 (17)	0.0644 (16)	0.0877 (19)	0.0018 (13)	0.0192 (14)	−0.0059 (14)
C37	0.094 (3)	0.057 (2)	0.077 (3)	−0.006 (2)	0.022 (2)	−0.005 (2)
C38	0.126 (5)	0.075 (3)	0.080 (3)	0.030 (3)	0.007 (3)	−0.010 (2)
C39	0.108 (4)	0.111 (4)	0.115 (4)	0.037 (3)	−0.021 (3)	−0.023 (3)
C40	0.061 (3)	0.090 (3)	0.138 (5)	0.001 (2)	0.020 (3)	−0.041 (3)

### Geometric parameters (Å, º)

**Table:**

Fe1—N3	1.9673 (17)	C23—H23A	0.9500
Fe1—N2	1.9675 (17)	C24—C25	1.382 (4)
Fe1—N4	1.9797 (18)	C24—H24A	0.9500
Fe1—N1	1.9853 (17)	C25—C27	1.371 (4)
Fe1—N7	2.0039 (17)	C25—C26	1.505 (3)
Fe1—N5	2.0078 (17)	C26—H26A	0.9800
N1—C102	1.375 (3)	C26—H26B	0.9800
N1—C101	1.376 (3)	C26—H26C	0.9800
N2—C103	1.372 (3)	C27—C28	1.390 (4)
N2—C104	1.378 (3)	C27—H27A	0.9500
N3—C106	1.377 (3)	C28—H28A	0.9500
N3—C105	1.378 (3)	C29—C30	1.339 (3)
N4—C108	1.376 (3)	C29—H29A	0.9500
N4—C107	1.377 (3)	C30—H30A	0.9500
N5—C31	1.326 (3)	C31—C32	1.470 (4)
N5—C29	1.403 (3)	C32—H32A	0.9800
N6—C31	1.340 (3)	C32—H32B	0.9800
N6—C30	1.370 (3)	C32—H32C	0.9800
N6—H6A	0.81 (3)	C33—C34	1.342 (3)
N7—C35	1.330 (3)	C33—H33A	0.9500
N7—C33	1.399 (3)	C34—H34A	0.9500
N8—C35	1.349 (3)	C35—C36	1.481 (3)
N8—C34	1.359 (3)	C36—H36A	0.9800
N8—H8A	0.84 (3)	C36—H36B	0.9800
C1—C2	1.391 (3)	C36—H36C	0.9800
C1—C7	1.393 (4)	C101—C301	1.396 (3)
C1—C301	1.496 (3)	C101—C201	1.442 (3)
C2—C3	1.390 (4)	C102—C302	1.385 (3)
C2—H2A	0.9500	C102—C202	1.442 (3)
C3—C4	1.379 (5)	C103—C302	1.397 (3)
C3—H3A	0.9500	C103—C203	1.432 (3)
C4—C6	1.389 (5)	C104—C303	1.395 (3)
C4—C5	1.519 (4)	C104—C204	1.436 (3)
C5—H5A	0.9800	C105—C303	1.395 (3)
C5—H5B	0.9800	C105—C205	1.436 (3)
C5—H5C	0.9800	C106—C304	1.391 (3)
C6—C7	1.390 (4)	C106—C206	1.440 (3)
C6—H6B	0.9500	C107—C304	1.389 (3)
C7—H7A	0.9500	C107—C207	1.435 (3)
C8—C14	1.370 (4)	C108—C301	1.397 (3)
C8—C9	1.394 (4)	C108—C208	1.439 (3)
C8—C302	1.494 (3)	C201—C202	1.346 (3)
C9—C10	1.395 (4)	C201—H20I	0.9500
C9—H9A	0.9500	C202—H20J	0.9500
C10—C11	1.380 (6)	C203—C204	1.347 (3)
C10—H10A	0.9500	C203—H20C	0.9500
C11—C13	1.364 (5)	C204—H20D	0.9500
C11—C12	1.522 (4)	C205—C206	1.351 (3)
C12—H12A	0.9800	C205—H20E	0.9500
C12—H12B	0.9800	C206—H20F	0.9500
C12—H12C	0.9800	C207—C208	1.349 (3)
C13—C14	1.395 (4)	C207—H20G	0.9500
C13—H13A	0.9500	C208—H20H	0.9500
C14—H14A	0.9500	O6—C44	1.456 (9)
C15—C16B	1.212 (11)	O6—C42	1.486 (9)
C15—C21B	1.345 (11)	C41—C42	1.459 (9)
C15—C21A	1.397 (4)	C41—C43	1.480 (9)
C15—C16A	1.403 (4)	C41—H41A	0.9900
C15—C303	1.496 (3)	C41—H41B	0.9900
C16A—C17A	1.387 (5)	C42—H42A	0.9900
C16A—H16A	0.9500	C42—H42B	0.9900
C17A—C18	1.375 (5)	C43—C44	1.456 (9)
C17A—H17A	0.9500	C43—H43A	0.9900
C20A—C21A	1.385 (5)	C43—H43B	0.9900
C20A—C18	1.406 (5)	C44—H44A	0.9900
C20A—H20A	0.9500	C44—H44B	0.9900
C21A—H21A	0.9500	Cl1—O1	1.423 (2)
C16B—C17B	1.540 (15)	Cl1—O4	1.425 (2)
C16B—H16B	0.9500	Cl1—O2	1.435 (2)
C17B—C18	1.476 (13)	Cl1—O3	1.438 (2)
C17B—H17B	0.9500	O5—C40	1.414 (5)
C20B—C18	1.255 (12)	O5—C37	1.421 (5)
C20B—C21B	1.409 (15)	C37—C38	1.487 (6)
C20B—H20B	0.9500	C37—H37A	0.9900
C21B—H21B	0.9500	C37—H37B	0.9900
C18—C19	1.513 (3)	C38—C39	1.488 (8)
C19—H19A	0.9800	C38—H38A	0.9900
C19—H19B	0.9800	C38—H38B	0.9900
C19—H19C	0.9800	C39—C40	1.530 (8)
C22—C28	1.384 (3)	C39—H39A	0.9900
C22—C23	1.385 (3)	C39—H39B	0.9900
C22—C304	1.493 (3)	C40—H40A	0.9900
C23—C24	1.388 (3)	C40—H40B	0.9900
			
N3—Fe1—N2	89.67 (7)	C22—C28—C27	121.0 (2)
N3—Fe1—N4	90.70 (7)	C22—C28—H28A	119.5
N2—Fe1—N4	179.46 (7)	C27—C28—H28A	119.5
N3—Fe1—N1	179.33 (7)	C30—C29—N5	109.3 (2)
N2—Fe1—N1	90.52 (7)	C30—C29—H29A	125.4
N4—Fe1—N1	89.11 (7)	N5—C29—H29A	125.4
N3—Fe1—N7	90.78 (7)	C29—C30—N6	106.0 (2)
N2—Fe1—N7	86.23 (7)	C29—C30—H30A	127.0
N4—Fe1—N7	93.38 (7)	N6—C30—H30A	127.0
N1—Fe1—N7	89.87 (7)	N5—C31—N6	109.7 (2)
N3—Fe1—N5	92.46 (7)	N5—C31—C32	128.8 (2)
N2—Fe1—N5	92.20 (7)	N6—C31—C32	121.4 (2)
N4—Fe1—N5	88.17 (7)	C31—C32—H32A	109.5
N1—Fe1—N5	86.90 (7)	C31—C32—H32B	109.5
N7—Fe1—N5	176.39 (7)	H32A—C32—H32B	109.5
C102—N1—C101	106.24 (17)	C31—C32—H32C	109.5
C102—N1—Fe1	125.50 (14)	H32A—C32—H32C	109.5
C101—N1—Fe1	127.79 (14)	H32B—C32—H32C	109.5
C103—N2—C104	105.79 (17)	C34—C33—N7	109.7 (2)
C103—N2—Fe1	126.90 (14)	C34—C33—H33A	125.1
C104—N2—Fe1	127.10 (14)	N7—C33—H33A	125.1
C106—N3—C105	106.54 (17)	C33—C34—N8	105.72 (19)
C106—N3—Fe1	126.38 (14)	C33—C34—H34A	127.1
C105—N3—Fe1	127.08 (14)	N8—C34—H34A	127.1
C108—N4—C107	105.90 (17)	N7—C35—N8	109.10 (19)
C108—N4—Fe1	128.30 (14)	N7—C35—C36	129.1 (2)
C107—N4—Fe1	125.70 (14)	N8—C35—C36	121.81 (19)
C31—N5—C29	105.87 (18)	C35—C36—H36A	109.5
C31—N5—Fe1	133.27 (16)	C35—C36—H36B	109.5
C29—N5—Fe1	120.58 (15)	H36A—C36—H36B	109.5
C31—N6—C30	109.1 (2)	C35—C36—H36C	109.5
C31—N6—H6A	126 (2)	H36A—C36—H36C	109.5
C30—N6—H6A	125 (2)	H36B—C36—H36C	109.5
C35—N7—C33	105.78 (17)	N1—C101—C301	126.07 (19)
C35—N7—Fe1	133.34 (15)	N1—C101—C201	109.70 (19)
C33—N7—Fe1	120.44 (14)	C301—C101—C201	123.9 (2)
C35—N8—C34	109.68 (19)	N1—C102—C302	125.87 (19)
C35—N8—H8A	124 (2)	N1—C102—C202	109.59 (19)
C34—N8—H8A	126 (2)	C302—C102—C202	124.4 (2)
C2—C1—C7	118.0 (2)	N2—C103—C302	125.3 (2)
C2—C1—C301	120.5 (2)	N2—C103—C203	110.10 (18)
C7—C1—C301	121.3 (2)	C302—C103—C203	124.3 (2)
C3—C2—C1	120.6 (3)	N2—C104—C303	125.02 (19)
C3—C2—H2A	119.7	N2—C104—C204	109.75 (18)
C1—C2—H2A	119.7	C303—C104—C204	124.9 (2)
C4—C3—C2	121.4 (3)	N3—C105—C303	125.62 (18)
C4—C3—H3A	119.3	N3—C105—C205	109.34 (18)
C2—C3—H3A	119.3	C303—C105—C205	124.82 (19)
C3—C4—C6	118.2 (3)	N3—C106—C304	125.42 (19)
C3—C4—C5	120.5 (3)	N3—C106—C206	109.43 (19)
C6—C4—C5	121.3 (3)	C304—C106—C206	124.84 (19)
C4—C5—H5A	109.5	N4—C107—C304	125.99 (19)
C4—C5—H5B	109.5	N4—C107—C207	109.75 (18)
H5A—C5—H5B	109.5	C304—C107—C207	124.17 (19)
C4—C5—H5C	109.5	N4—C108—C301	125.5 (2)
H5A—C5—H5C	109.5	N4—C108—C208	109.94 (18)
H5B—C5—H5C	109.5	C301—C108—C208	124.2 (2)
C4—C6—C7	120.9 (3)	C202—C201—C101	107.1 (2)
C4—C6—H6B	119.6	C202—C201—H20I	126.4
C7—C6—H6B	119.6	C101—C201—H20I	126.4
C6—C7—C1	120.9 (3)	C201—C202—C102	107.3 (2)
C6—C7—H7A	119.6	C201—C202—H20J	126.3
C1—C7—H7A	119.6	C102—C202—H20J	126.3
C14—C8—C9	118.9 (2)	C204—C203—C103	107.2 (2)
C14—C8—C302	119.6 (2)	C204—C203—H20C	126.4
C9—C8—C302	121.4 (2)	C103—C203—H20C	126.4
C8—C9—C10	119.4 (3)	C203—C204—C104	107.1 (2)
C8—C9—H9A	120.3	C203—C204—H20D	126.4
C10—C9—H9A	120.3	C104—C204—H20D	126.4
C11—C10—C9	121.3 (3)	C206—C205—C105	107.56 (19)
C11—C10—H10A	119.4	C206—C205—H20E	126.2
C9—C10—H10A	119.4	C105—C205—H20E	126.2
C13—C11—C10	118.6 (3)	C205—C206—C106	107.07 (19)
C13—C11—C12	119.1 (4)	C205—C206—H20F	126.5
C10—C11—C12	122.3 (4)	C106—C206—H20F	126.5
C11—C12—H12A	109.5	C208—C207—C107	107.46 (19)
C11—C12—H12B	109.5	C208—C207—H20G	126.3
H12A—C12—H12B	109.5	C107—C207—H20G	126.3
C11—C12—H12C	109.5	C207—C208—C108	106.85 (19)
H12A—C12—H12C	109.5	C207—C208—H20H	126.6
H12B—C12—H12C	109.5	C108—C208—H20H	126.6
C11—C13—C14	120.9 (3)	C101—C301—C108	122.2 (2)
C11—C13—H13A	119.5	C101—C301—C1	116.95 (19)
C14—C13—H13A	119.5	C108—C301—C1	120.2 (2)
C8—C14—C13	120.8 (3)	C102—C302—C103	123.4 (2)
C8—C14—H14A	119.6	C102—C302—C8	117.39 (19)
C13—C14—H14A	119.6	C103—C302—C8	119.2 (2)
C16B—C15—C21B	118.6 (6)	C105—C303—C104	122.67 (19)
C21A—C15—C16A	118.7 (3)	C105—C303—C15	118.52 (18)
C16B—C15—C303	117.7 (5)	C104—C303—C15	118.34 (19)
C21B—C15—C303	123.6 (5)	C107—C304—C106	123.26 (19)
C21A—C15—C303	119.8 (2)	C107—C304—C22	117.68 (19)
C16A—C15—C303	121.5 (2)	C106—C304—C22	118.87 (18)
C17A—C16A—C15	119.9 (3)	C44—O6—C42	104.2 (8)
C17A—C16A—H16A	120.1	C42—C41—C43	100.2 (9)
C15—C16A—H16A	120.1	C42—C41—H41A	111.7
C18—C17A—C16A	122.1 (3)	C43—C41—H41A	111.7
C18—C17A—H17A	119.0	C42—C41—H41B	111.7
C16A—C17A—H17A	119.0	C43—C41—H41B	111.7
C21A—C20A—C18	121.0 (3)	H41A—C41—H41B	109.5
C21A—C20A—H20A	119.5	C41—C42—O6	107.7 (9)
C18—C20A—H20A	119.5	C41—C42—H42A	110.2
C20A—C21A—C15	120.3 (3)	O6—C42—H42A	110.2
C20A—C21A—H21A	119.8	C41—C42—H42B	110.2
C15—C21A—H21A	119.8	O6—C42—H42B	110.2
C15—C16B—C17B	123.0 (9)	H42A—C42—H42B	108.5
C15—C16B—H16B	118.5	C44—C43—C41	102.9 (8)
C17B—C16B—H16B	118.5	C44—C43—H43A	111.2
C18—C17B—C16B	112.4 (8)	C41—C43—H43A	111.2
C18—C17B—H17B	123.8	C44—C43—H43B	111.2
C16B—C17B—H17B	123.8	C41—C43—H43B	111.2
C18—C20B—C21B	123.1 (9)	H43A—C43—H43B	109.1
C18—C20B—H20B	118.5	C43—C44—O6	105.2 (8)
C21B—C20B—H20B	118.5	C43—C44—H44A	110.7
C15—C21B—C20B	122.4 (9)	O6—C44—H44A	110.7
C15—C21B—H21B	118.8	C43—C44—H44B	110.7
C20B—C21B—H21B	118.8	O6—C44—H44B	110.7
C17A—C18—C20A	117.9 (3)	H44A—C44—H44B	108.8
C20B—C18—C17B	117.5 (7)	O1—Cl1—O4	109.59 (17)
C20B—C18—C19	121.4 (5)	O1—Cl1—O2	110.10 (18)
C17A—C18—C19	121.5 (3)	O4—Cl1—O2	107.68 (12)
C20A—C18—C19	120.5 (3)	O1—Cl1—O3	109.86 (13)
C17B—C18—C19	120.5 (5)	O4—Cl1—O3	109.91 (14)
C18—C19—H19A	109.5	O2—Cl1—O3	109.67 (15)
C18—C19—H19B	109.5	C40—O5—C37	103.8 (4)
H19A—C19—H19B	109.5	O5—C37—C38	106.6 (4)
C18—C19—H19C	109.5	O5—C37—H37A	110.4
H19A—C19—H19C	109.5	C38—C37—H37A	110.4
H19B—C19—H19C	109.5	O5—C37—H37B	110.4
C28—C22—C23	117.8 (2)	C38—C37—H37B	110.4
C28—C22—C304	120.6 (2)	H37A—C37—H37B	108.6
C23—C22—C304	121.57 (19)	C37—C38—C39	104.7 (4)
C22—C23—C24	120.6 (2)	C37—C38—H38A	110.8
C22—C23—H23A	119.7	C39—C38—H38A	110.8
C24—C23—H23A	119.7	C37—C38—H38B	110.8
C25—C24—C23	121.3 (2)	C39—C38—H38B	110.8
C25—C24—H24A	119.4	H38A—C38—H38B	108.9
C23—C24—H24A	119.4	C38—C39—C40	103.8 (4)
C27—C25—C24	118.1 (2)	C38—C39—H39A	111.0
C27—C25—C26	120.7 (3)	C40—C39—H39A	111.0
C24—C25—C26	121.2 (3)	C38—C39—H39B	111.0
C25—C26—H26A	109.5	C40—C39—H39B	111.0
C25—C26—H26B	109.5	H39A—C39—H39B	109.0
H26A—C26—H26B	109.5	O5—C40—C39	106.4 (4)
C25—C26—H26C	109.5	O5—C40—H40A	110.5
H26A—C26—H26C	109.5	C39—C40—H40A	110.5
H26B—C26—H26C	109.5	O5—C40—H40B	110.5
C25—C27—C28	121.1 (2)	C39—C40—H40B	110.5
C25—C27—H27A	119.5	H40A—C40—H40B	108.6
C28—C27—H27A	119.5		
			
C7—C1—C2—C3	−1.0 (4)	C105—N3—C106—C206	−1.1 (2)
C301—C1—C2—C3	173.9 (2)	Fe1—N3—C106—C206	177.96 (13)
C1—C2—C3—C4	0.5 (4)	C108—N4—C107—C304	−178.7 (2)
C2—C3—C4—C6	0.2 (4)	Fe1—N4—C107—C304	4.7 (3)
C2—C3—C4—C5	−179.5 (3)	C108—N4—C107—C207	−2.0 (2)
C3—C4—C6—C7	−0.2 (4)	Fe1—N4—C107—C207	−178.55 (13)
C5—C4—C6—C7	179.4 (3)	C107—N4—C108—C301	−169.7 (2)
C4—C6—C7—C1	−0.4 (4)	Fe1—N4—C108—C301	6.8 (3)
C2—C1—C7—C6	1.0 (4)	C107—N4—C108—C208	3.2 (2)
C301—C1—C7—C6	−173.9 (2)	Fe1—N4—C108—C208	179.60 (14)
C14—C8—C9—C10	−0.3 (4)	N1—C101—C201—C202	1.9 (3)
C302—C8—C9—C10	−176.4 (3)	C301—C101—C201—C202	−171.4 (2)
C8—C9—C10—C11	1.4 (5)	C101—C201—C202—C102	−2.0 (3)
C9—C10—C11—C13	−1.9 (5)	N1—C102—C202—C201	1.5 (3)
C9—C10—C11—C12	175.7 (3)	C302—C102—C202—C201	−174.1 (2)
C10—C11—C13—C14	1.4 (5)	N2—C103—C203—C204	−1.7 (3)
C12—C11—C13—C14	−176.3 (3)	C302—C103—C203—C204	172.7 (2)
C9—C8—C14—C13	−0.2 (4)	C103—C203—C204—C104	2.5 (3)
C302—C8—C14—C13	175.9 (3)	N2—C104—C204—C203	−2.5 (3)
C11—C13—C14—C8	−0.3 (5)	C303—C104—C204—C203	171.0 (2)
C21A—C15—C16A—C17A	2.0 (5)	N3—C105—C205—C206	1.7 (2)
C303—C15—C16A—C17A	179.5 (3)	C303—C105—C205—C206	−173.1 (2)
C15—C16A—C17A—C18	0.5 (6)	C105—C205—C206—C106	−2.3 (2)
C18—C20A—C21A—C15	−1.4 (6)	N3—C106—C206—C205	2.2 (2)
C16A—C15—C21A—C20A	−1.5 (5)	C304—C106—C206—C205	−171.6 (2)
C303—C15—C21A—C20A	−179.1 (3)	N4—C107—C207—C208	0.0 (2)
C21B—C15—C16B—C17B	14.2 (17)	C304—C107—C207—C208	176.8 (2)
C303—C15—C16B—C17B	−168.8 (9)	C107—C207—C208—C108	1.9 (2)
C15—C16B—C17B—C18	−20.5 (17)	N4—C108—C208—C207	−3.2 (2)
C16B—C15—C21B—C20B	−4.2 (18)	C301—C108—C208—C207	169.7 (2)
C303—C15—C21B—C20B	178.9 (10)	N1—C101—C301—C108	−7.2 (3)
C18—C20B—C21B—C15	2 (2)	C201—C101—C301—C108	165.0 (2)
C21B—C20B—C18—C17B	−9.2 (18)	N1—C101—C301—C1	−178.28 (19)
C21B—C20B—C18—C19	−179.9 (11)	C201—C101—C301—C1	−6.0 (3)
C16A—C17A—C18—C20A	−3.3 (5)	N4—C108—C301—C101	3.8 (3)
C16A—C17A—C18—C19	179.1 (3)	C208—C108—C301—C101	−168.1 (2)
C21A—C20A—C18—C17A	3.8 (6)	N4—C108—C301—C1	174.58 (19)
C21A—C20A—C18—C19	−178.6 (3)	C208—C108—C301—C1	2.7 (3)
C16B—C17B—C18—C20B	16.8 (15)	C2—C1—C301—C101	−63.6 (3)
C16B—C17B—C18—C19	−172.5 (8)	C7—C1—C301—C101	111.2 (3)
C28—C22—C23—C24	1.6 (4)	C2—C1—C301—C108	125.2 (2)
C304—C22—C23—C24	−179.9 (2)	C7—C1—C301—C108	−60.1 (3)
C22—C23—C24—C25	0.6 (4)	N1—C102—C302—C103	−3.6 (3)
C23—C24—C25—C27	−1.9 (4)	C202—C102—C302—C103	171.2 (2)
C23—C24—C25—C26	176.9 (2)	N1—C102—C302—C8	179.2 (2)
C24—C25—C27—C28	1.1 (4)	C202—C102—C302—C8	−6.0 (3)
C26—C25—C27—C28	−177.8 (3)	N2—C103—C302—C102	9.7 (3)
C23—C22—C28—C27	−2.4 (4)	C203—C103—C302—C102	−163.9 (2)
C304—C22—C28—C27	179.1 (2)	N2—C103—C302—C8	−173.1 (2)
C25—C27—C28—C22	1.1 (4)	C203—C103—C302—C8	13.4 (3)
C31—N5—C29—C30	−0.4 (3)	C14—C8—C302—C102	−97.3 (3)
Fe1—N5—C29—C30	174.29 (19)	C9—C8—C302—C102	78.8 (3)
N5—C29—C30—N6	0.6 (3)	C14—C8—C302—C103	85.3 (3)
C31—N6—C30—C29	−0.6 (3)	C9—C8—C302—C103	−98.6 (3)
C29—N5—C31—N6	0.0 (3)	N3—C105—C303—C104	−7.3 (3)
Fe1—N5—C31—N6	−173.71 (16)	C205—C105—C303—C104	166.6 (2)
C29—N5—C31—C32	−178.4 (3)	N3—C105—C303—C15	−179.31 (19)
Fe1—N5—C31—C32	7.9 (4)	C205—C105—C303—C15	−5.4 (3)
C30—N6—C31—N5	0.4 (3)	N2—C104—C303—C105	3.1 (3)
C30—N6—C31—C32	179.0 (3)	C204—C104—C303—C105	−169.5 (2)
C35—N7—C33—C34	−0.1 (2)	N2—C104—C303—C15	175.08 (19)
Fe1—N7—C33—C34	−173.43 (15)	C204—C104—C303—C15	2.5 (3)
N7—C33—C34—N8	0.4 (3)	C16B—C15—C303—C105	−108.5 (8)
C35—N8—C34—C33	−0.5 (3)	C21B—C15—C303—C105	68.4 (9)
C33—N7—C35—N8	−0.3 (2)	C21A—C15—C303—C105	110.0 (3)
Fe1—N7—C35—N8	171.86 (15)	C16A—C15—C303—C105	−67.5 (3)
C33—N7—C35—C36	−179.5 (2)	C16B—C15—C303—C104	79.2 (8)
Fe1—N7—C35—C36	−7.4 (4)	C21B—C15—C303—C104	−103.9 (9)
C34—N8—C35—N7	0.5 (3)	C21A—C15—C303—C104	−62.3 (3)
C34—N8—C35—C36	179.8 (2)	C16A—C15—C303—C104	120.2 (3)
C102—N1—C101—C301	172.2 (2)	N4—C107—C304—C106	8.8 (3)
Fe1—N1—C101—C301	−0.1 (3)	C207—C107—C304—C106	−167.5 (2)
C102—N1—C101—C201	−0.9 (2)	N4—C107—C304—C22	−176.28 (19)
Fe1—N1—C101—C201	−173.29 (14)	C207—C107—C304—C22	7.4 (3)
C101—N1—C102—C302	175.2 (2)	N3—C106—C304—C107	−7.0 (3)
Fe1—N1—C102—C302	−12.3 (3)	C206—C106—C304—C107	165.9 (2)
C101—N1—C102—C202	−0.3 (2)	N3—C106—C304—C22	178.18 (19)
Fe1—N1—C102—C202	172.27 (14)	C206—C106—C304—C22	−9.0 (3)
C104—N2—C103—C302	−174.2 (2)	C28—C22—C304—C107	79.0 (3)
Fe1—N2—C103—C302	0.9 (3)	C23—C22—C304—C107	−99.4 (3)
C104—N2—C103—C203	0.2 (2)	C28—C22—C304—C106	−105.8 (3)
Fe1—N2—C103—C203	175.23 (14)	C23—C22—C304—C106	75.7 (3)
C103—N2—C104—C303	−172.1 (2)	C43—C41—C42—O6	32.1 (17)
Fe1—N2—C104—C303	12.8 (3)	C44—O6—C42—C41	−8.5 (19)
C103—N2—C104—C204	1.4 (2)	C42—C41—C43—C44	−43.7 (14)
Fe1—N2—C104—C204	−173.66 (14)	C41—C43—C44—O6	39.9 (15)
C106—N3—C105—C303	174.41 (19)	C42—O6—C44—C43	−19.3 (18)
Fe1—N3—C105—C303	−4.6 (3)	C40—O5—C37—C38	−39.0 (4)
C106—N3—C105—C205	−0.3 (2)	O5—C37—C38—C39	26.3 (5)
Fe1—N3—C105—C205	−179.38 (14)	C37—C38—C39—C40	−4.1 (5)
C105—N3—C106—C304	172.64 (19)	C37—O5—C40—C39	35.8 (5)
Fe1—N3—C106—C304	−8.3 (3)	C38—C39—C40—O5	−19.2 (5)

### Hydrogen-bond geometry (Å, º)

**Table:**

*D*—H···*A*	*D*—H	H···*A*	*D*···*A*	*D*—H···*A*
N6—H6*A*···O3^i^	0.81 (3)	2.17 (3)	2.942 (3)	161 (3)
N8—H8*A*···O2^ii^	0.84 (3)	2.11 (3)	2.949 (3)	176 (3)

Symmetry codes: (i) −*x*+1/2, *y*+1/2, −*z*+1/2; (ii) −*x*, *y*, −*z*+1/2.
